# Treatment of gastrointestinal tumor (GIST) of the rectum requiring abdominoperineal resection following neoadjuvant imatinib: a cost-effectiveness analysis

**DOI:** 10.1186/s13569-020-00135-7

**Published:** 2020-08-06

**Authors:** Mohamad Farid, Johnny Ong, Claramae Chia, Grace Tan, Melissa Teo, Richard Quek, Jonathan Teh, David Matchar

**Affiliations:** 1 Division of Medical Oncology, National Cancer Centre Singapore, 11 Hospital Drive, Singapore, 169610 Singapore; 2 Division of Surgical Oncology, National Cancer Centre, Singapore, Singapore; 3 Parkway Cancer Centre, Singapore, Singapore; 4 Radiation Oncology, Farrer Park Hospital, Singapore, Singapore; 5 Programme in Health Services and Systems Research, Graduate Medical School, Duke-National University of Singapore, Singapore, Singapore

**Keywords:** Rectal GIST treatment, Imatinib, Cost effectiveness analysis

## Abstract

**Background:**

Neoadjuvant imatinib for gastrointestinal stromal tumors (GIST) of the rectum can reduce, but may not eliminate, risk of surgical morbidity from permanent bowel diversion. We sought to evaluate the cost-effectiveness of alternative strategies in rectal GIST patients requiring abdominoperineal resection following neoadjuvant imatinib.

**Methods:**

We developed a Markov model using a healthcare payers’ perspective to estimate costs in 2017 Singapore dollars (SGD) and quality adjusted life years (QALYs) for upfront abdominoperineal resection (UAPR) versus continued imatinib until progression (CIUP) following 1 year of neoadjuvant imatinib. Transition probabilities and utilities were obtained from published data, and costs were estimated using data from the National Cancer Centre Singapore. Deterministic and probabilistic sensitivity analyses were conducted to probe model uncertainty. Incremental cost-effectiveness ratio below SGD 50,000 per QALY gained was considered cost-effective.

**Results:**

In the base case, UAPR dominates CIUP being both more effective (8.66 QALYS vs 5.43 QALYs) and less expensive (SGD 312,627 vs SGD 339,011). These estimates were most sensitive to 2 variables, utility of abdominoperineal resection and annual recurrence probability post-abdominoperineal resection. However, simultaneously varying the values of these variables to maximally favor CIUP did not render it the more cost effective strategy at willingness to pay (WTP) of SGD 50,000. In probabilistic sensitivity analysis, UAPR had probability of being cost-effective compared with CIUP greater than 95%, reaching 100% at WTP SGD 10,000.

**Conclusion:**

UAPR is more effective and less costly than CIUP for patients with rectal GIST requiring abdominoperineal resection following neoadjuvant imatinib, and is the strategy of choice in this setting.

## Background

Gastrointestinal stromal tumors (GIST) are mesenchymal tumors arising from the interstitial cells of Cajal in the alimentary tract with an incidence of 10–15 per million per year [1]. The vast majority of cases arise in the stomach (40–60%), or jejunum/ileum (25–30%); approximately 5–15% of cases arise from the colon or rectum [2]. The primary treatment of GIST is complete surgical resection; among individuals without high-risk clinicopathologic features for recurrence this approach results in sustained disease control in up to 70% of cases [3]. Imatinib, the tyrosine kinase inhibitor antagonizing the KIT and PDGFR oncoproteins implicated in the majority of GISTs, has revolutionized the treatment of this previously dismal disease. In advanced disease, imatinib achieves objective response rates, progression free survival (PFS) and overall survival (OS) of 50%, 1.5 years and 4 years, respectively [4]. In addition, 3 year of adjuvant imatinib improves overall survival in patients with high-risk localized disease who have undergone resection [5]. The achievement of this significant therapeutic advance has come with the convenience of a pill taken once daily having an excellent therapeutic index and minimal cumulative toxicity [6]. GISTs arising from the rectum poses a therapeutic challenge in view of potential for significant surgical morbidity from loss of bowel-sphincteric control and need for permanent bowel diversion if abdominoperineal resection is required for complete resection [7]. Neoadjuvant imatinib has been shown to be safe and efficacious in the treatment of GIST presenting challenges to upfront surgery, including disease arising from the rectum [8, 9]. Expert guidelines suggest that neoadjuvant imatinib be given for up to 12 months in such cases to maximize response prior to surgery, with consideration given for more imatinib postoperatively to achieve a total of 3 years of imatinib therapy [10]. Although surgical resection remains the cornerstone of curative therapy, some patients continue to defer curative surgery at the end of this period in view of the excellent therapeutic index of imatinib and fear of permanent morbidity from APR, the need for which may persist even after maximal tumor response to neoadjuvant imatinib. These patients may elect to continue imatinib indefinitely in lieu of upfront surgery, to avoid surgical morbidity unless absolutely necessary, if and when they progress on imatinib. Notwithstanding the excellent therapeutic index of imatinib, there are no data to suggest that indefinite imatinib can substitute surgery as curative treatment in localized disease. The natural history for patients receiving imatinib in advanced disease is the eventual development of resistance and consequent local as well as distant progression. The development of metastatic disease precludes therapy with curative intent and leads to eventual morbidity and mortality [11]. While imatinib has been shown to be cost-effective in both the adjuvant [12] and advanced [13] disease setting, there are no data on the cost-effectiveness of imatinib as primary therapy in a setting where upfront surgery has significant potential morbidity, specifically in rectal GIST.

We sought to estimate, using a Markov decision model, the relative effectiveness and cost-effectiveness of upfront abdominoperineal resection compared with continued imatinib until progression for patients with rectal GIST having received neoadjuvant imatinib who would require abdominoperineal resection for surgical extirpation.

## Methods

### Theoretical model/model overview

**Fig. 1:**
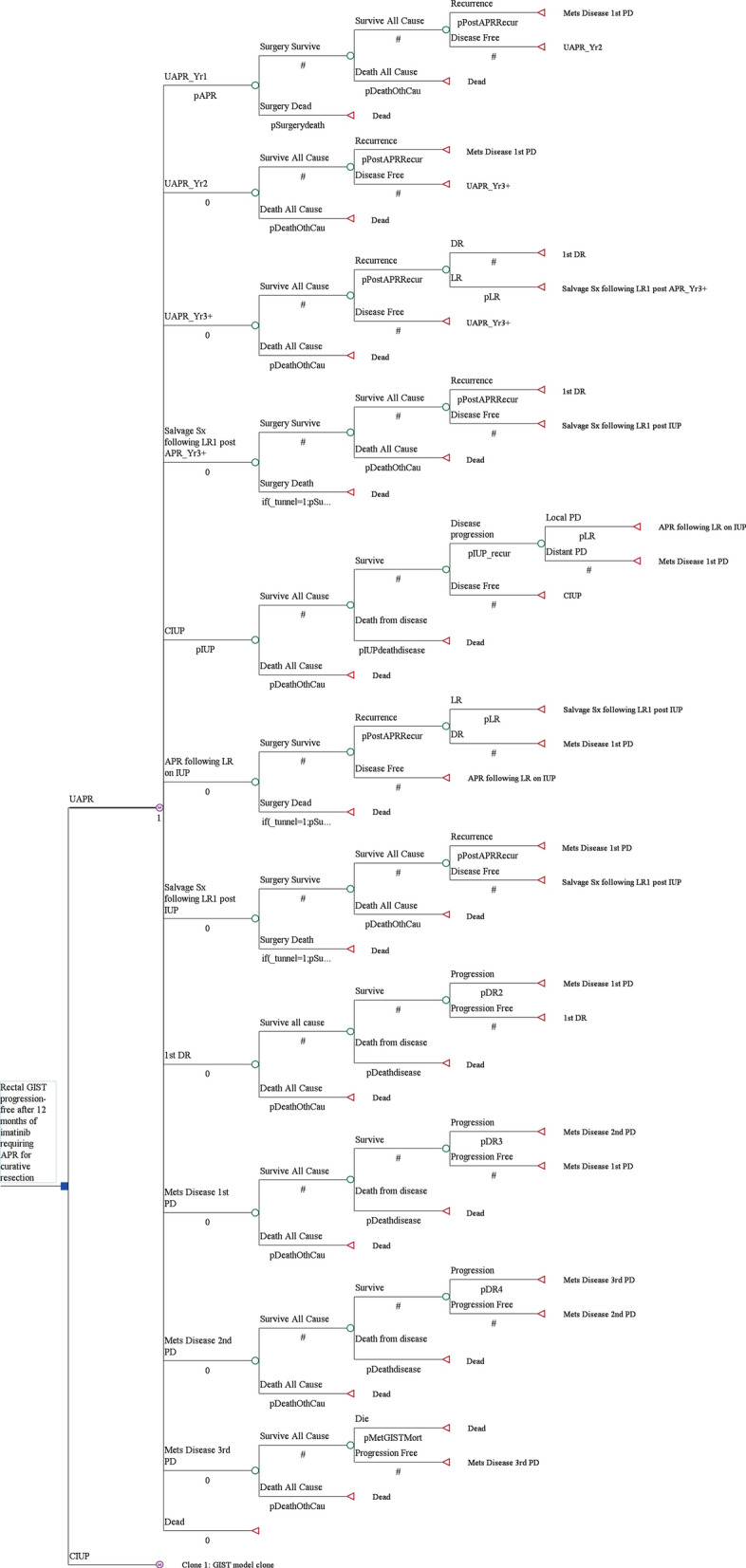
Markov model structure evaluating 2 treatment strategies (UAPR and CIUP) with 12 health states

A comprehensive Markov model was developed to analyze the health and cost impact of alternative strategies in management of rectal GIST from a healthcare sectors’ perspective in Singapore. The structure of a Markov model is composed of mutually exclusive health states relevant to the impact of disease and treatments under evaluation. Over a pre-determined time horizon, any patient can stay in only 1 health state at any time, but may move between health states over time [14]. We modelled a hypothetical cohort of 60-year old patients with rectal GIST who had received 1 year of neoadjuvant imatinib at 400 milligrams daily with no disease progression, for which resection would necessitate an abdominoperineal resection. The model evaluated 2 treatment options - continued imatinib until progression (CIUP), or surgical resection with upfront abdominoperineal resection (UAPR). (Figure 1) The model had a cycle length of 1 year and followed patients over a time horizon of 20 years. A total of 12 health states were defined: UAPR at 1st year (“UAPR_Yr1”), UAPR at 2nd year (“UAPR_Yr2”) and UAPR at 3rd year and beyond (“UAPR_Yr3+”) represent patients at different time-points following upfront abdominoperineal resection. The fourth health state accounts for patients receiving salvage surgery for their 1st local recurrence following upfront abdominoperineal resection (“Salvage Sx following LR1 post UAPR_Yr3+”). The fifth health state addresses patients receiving the second strategy, CIUP (“CIUP”). The sixth and seventh health states contains patients following local progression on CIUP–either undergoing abdominoperineal resection following local progression on CIUP (“abdominoperineal resection following LR on CIUP”) or subsequently salvage surgery following 1st local recurrence after abdominoperineal resection on CIUP (“Salvage Sx following LR1 post CIUP”). The eighth through eleventh health states encompasses patients at different lines of palliative therapy in advanced disease, namely: distant recurrence (“1st DR”) where they receive imatinib 400 milligrams daily [11]; 1st progression in metastatic disease (“mets disease 1st PD”) where they receive imatinib 800 milligrams daily [11]; 2nd progression in metastatic disease (“mets disease 2nd PD”) where they receive sunitinib 50 milligrams daily 4 weeks on and 2 weeks off in a 6-weekly cycle [15]; and 3rd progression in metastatic disease (“mets disease 3rd PD”) where they receive regorafenib 160 milligrams daily 3 weeks on and 1 week off in a 4-weekly cycle [16]. The twelfth and final health state represents death (“Dead”).

For patients on UAPR, they received 2 years of postoperative imatinib, to make up a total of 3 years of neoadjuvant and adjuvant imatinib, as consistent with treatment guidelines [10]. Patients with disease progression while on adjuvant imatinib have a dismal prognosis, and thus transit directly to the health state where they receive escalated doses of imatinib, “mets disease 1st PD”, regardless of whether the progression was local or distant. The first local recurrence after patients complete 2 years of adjuvant imatinib therapy can yet be treated with salvage surgery followed by 3 further years of adjuvant imatinib, a strategy with demonstrated benefit in newly diagnosed high-risk resected GIST [5]. The second local recurrence or distant recurrence would lead to patients entering the metastatic disease health state receiving first line palliative therapy with imatinib, ‘1st DR’.

For patients on CIUP, local progression would be treated with abdominoperineal resection followed by no further Imatinib. The first local recurrence following such an abdominoperineal resection would be treated with salvage surgery only. The second local recurrence or distant recurrence would lead to patients directly entering the advanced disease health state receiving escalated doses of imatinib (“mets disease 1st PD”), as these patients have demonstrated failure to treatment with imatinib 400 milligrams daily. Tunnels states were used to facilitate the undertaking of salvage surgery in the setting of local recurrence (following APR in both the APR and IUP arms) only once.

Model outcomes were defined as treatment costs (in 2017 SGD) and quality-adjusted life years (QALYs). We calculated the incremental cost-effectiveness ratios (ICERs) of the more expensive to the less expensive strategy as the difference in costs divided by the difference in QALYs – to compare 2 treatment strategies. All costs and health outcomes were discounted by 3% annually. We adopted a willingness to pay (WTP) and ICER of SGD 50,000 per QALY gained as the threshold for cost-effectiveness [17]. The model was developed using the decision analytic software TreeAge Pro 2017 (TreeAge Software, Williamstown, MA).

### Model transitions and survival estimates

Event rates for progression and mortality were used to calculate transition probabilities [18, 19]. For advanced GIST, data was obtained from randomized controlled trials in the first [4], second [15] and third [16] line setting for GIST treatment. Recurrence rates following neoadjuvant imatinib and surgery for localized GIST were obtained from a large retrospective study of neoadjuvant imatinib in GIST [8]. Although there has been a prospective single arm phase 2 study of neoadjuvant imatinib in localized GIST [9], the perioperative imatinib regimen in the former study much more closely resembled that used in our model (12 months of imatinib at 400 milligrams daily), as opposed to the 2 months of 600 milligrams daily neoadjuvant imatinib employed in the RTOG 0132 study [9]. In addition, there were 33 patients with rectal GIST in the retrospective study, as opposed to only 3 with rectal disease in RTOG 0132. For these reasons, we decided to use data obtained from the retrospective study in our model. The range of values considered for these transition probabilities were derived from the confidence intervals reported in the literature or estimated if published data were unavailable (Table 1).

**Table 1:** Transition probabilities, utilities and costs

Variable	Base case	Range for sensitivity analysis	Distribution for probabilistic sensitivity analysis	Reference
Probabilities				
Annual probability of recurrence post abdominoperineal resection	0.0871	0.0435–0.130^a^	Beta	Rutkowski et al. [8]
Annual conditional probability of local recurrence post abdominoperineal resection^a^	0.135	0.0675–0.203^a^	Uniform	Rutkowski et al. [8]
Annual probability of 1st progression in metastatic GIST	0.370	0.296–0.444^b^	Beta	Blanke et al. [4]
Annual probability of 2nd progression in metastatic GIST	0.811	0.649–0.973^b^	Beta	Blanke et al. [4]
Annual probability of 3rd progression in metastatic GIST	0.708	0.566–0.850^b^	Beta	Demetri et al. [13]
Annual probability of death in metastatic GIST post-regorafenib	0.405	0.270–0.410^b^	Beta	Demetri et al. [14]
Utilities				
Recurrence-free health state post abdominoperineal resection	0.830	0.650–1	Beta	Miller et al. [7]
Recurrence-free health state on continued imatinib until progression	0.935	0.750–1^b^	Beta	Wilson et al. [17]
GIST recurrence	0.748	0.598–0.898^b^	Beta	Majer et al. [10]
GIST 1st progression in metastatic disease	0.712	0.685–0.739	Beta	Chabot et al. [18]
GIST 2nd progression in metastatic disease	0.712	0.685–0.739	Beta	Assumption
GIST 3rd progression in metastatic disease	0.712	0.685–0.739	Beta	Assumption
COSTS (SGD)				
Annual cost of imatinib 400 mg once daily	37 040	7 408–44 448^c^	Gamma	NCCS data
Annual cost of sunitinib 50 mg once daily 4 weeks on, 2 weeks off	64 063	51 250–76 876^b^	Gamma	NCCS data
Annual cost of regorafenib 160 mg once daily 3 weeks on, 1 week off	72 001	57 601–86 401^b^	Gamma	NCCS data
Abdominoperineal resection	38 000	30 400 –45 600^b^	Gamma	NCCS data
Salvage surgery (following 1st local recurrence post abdominoperineal resection)	38 000	30 400–45 600^b^	Gamma	NCCS data
Annual cost of follow-up (consultation, blood tests, computer tomography scans every 3 months)	3 000	2 400–3 600^b^	Gamma	NCCS data

*mg* milligrams, *GIST* gastrointestinal stromal tumor, *NCCS* National Cancer Centre Singapore, *SGD* Singapore dollars

^a^ ± 50% (wider intervals used in estimation due to paucity of systematic data for recurrences specifically in rectal GIST post neoadjuvant imatinib)

^b^ ± 20%

^c^ + 20% and −80% (lower bound for imatinib extended to −80% to account for possible significant decrease in imatinib cost with advent of generic imatinib)

The model simulated transitions between health states with a cycle length of 1 year, which was chosen as a clinically and therapeutically relevant interval for evaluation, considering the duration of GIST neoadjuvant and adjuvant (1 and 2 years, respectively) treatment and average duration of disease control. The probability of transitioning to death during each cycle was defined as the maximum value of observed mortality rate by using survival data from studies in GIST as well as the background mortality rate, estimated from age-specific death rates in Singapore [20]. The mortality of abdominoperineal resection and subsequent salvage surgery for first post-abdominoperineal resection local recurrence was assumed to be 0.5%.

### Utilities and costs

QALYs were calculated by multiplying the time spent in a given state (in life-years) by the utility score (a health status value ranging from 0 for death to 1 for perfect health) associated with that state. The utility weights of all health states were derived from published studies (Table 1). Utility associated with abdominoperineal resection was obtained from studies performed in recurrent rectal cancer [7]. Costs of surgery were estimated based on data from the National Cancer Centre Singapore (NCCS). Drug costs were derived from the NCCS pharmacy data. Clinical consultation, blood tests and computed tomography (CT) scan performed every 3 monthly as surveillance were estimated to cost 3000 SGD per year based on expert opinion and NCCS data.

### Sensitivity analysis

To evaluate the robustness of the model and address the uncertainty in estimation of variables, we performed a series of one-way deterministic sensitivity analyses (DSA), in each instance varying the value of one parameter at a time over its defined range and examining the effect of each parameter individually on ICERs for all variables. Where available, the range of values considered for these sensitivity analyses were derived from the confidence intervals reported in the primary literature (Table 1).

To account for variation in multiple parameters simultaneously, we also completed probabilistic sensitivity analyses (PSA). We performed 10,000 Monte Carlo simulations, each time randomly sampling from the distributions for all parameters simultaneously. Uncertainty in utilities was represented by β distributions which are bounded by 0 and 1, while uncertainty in cost was represented by $\gamma$ distributions, which are bounded by 0 and infinity [21]. To succinctly represent uncertainty surrounding our base case estimate of cost-effectiveness, cost-effectiveness acceptability curves were derived and used to project the probability that each treatment strategy was economically preferred under various WTP thresholds.

## Results

### Base case/main analysis

Examining the 2 treatment strategies over the 20-year time horizon, UAPR (SGD 312,627) is less costly compared to CIUP (SGD 339,011), yet generates more QALYs (8.66 vs 5.43 years), thus dominating over CIUP as a strategy.

### Sensitivity analysis

One-way DSA revealed our results to be robust for all variables across the pre-defined range of values (Table 1), with UAPR consistently dominating over CIUP; the only exception to this arose in the context of annual recurrence probability post abdominoperineal resection greater than 12.5%, where an ICER for UAPR emerged, though remaining well below SGD 50,000 per QALY gained.

**Fig. 2:**
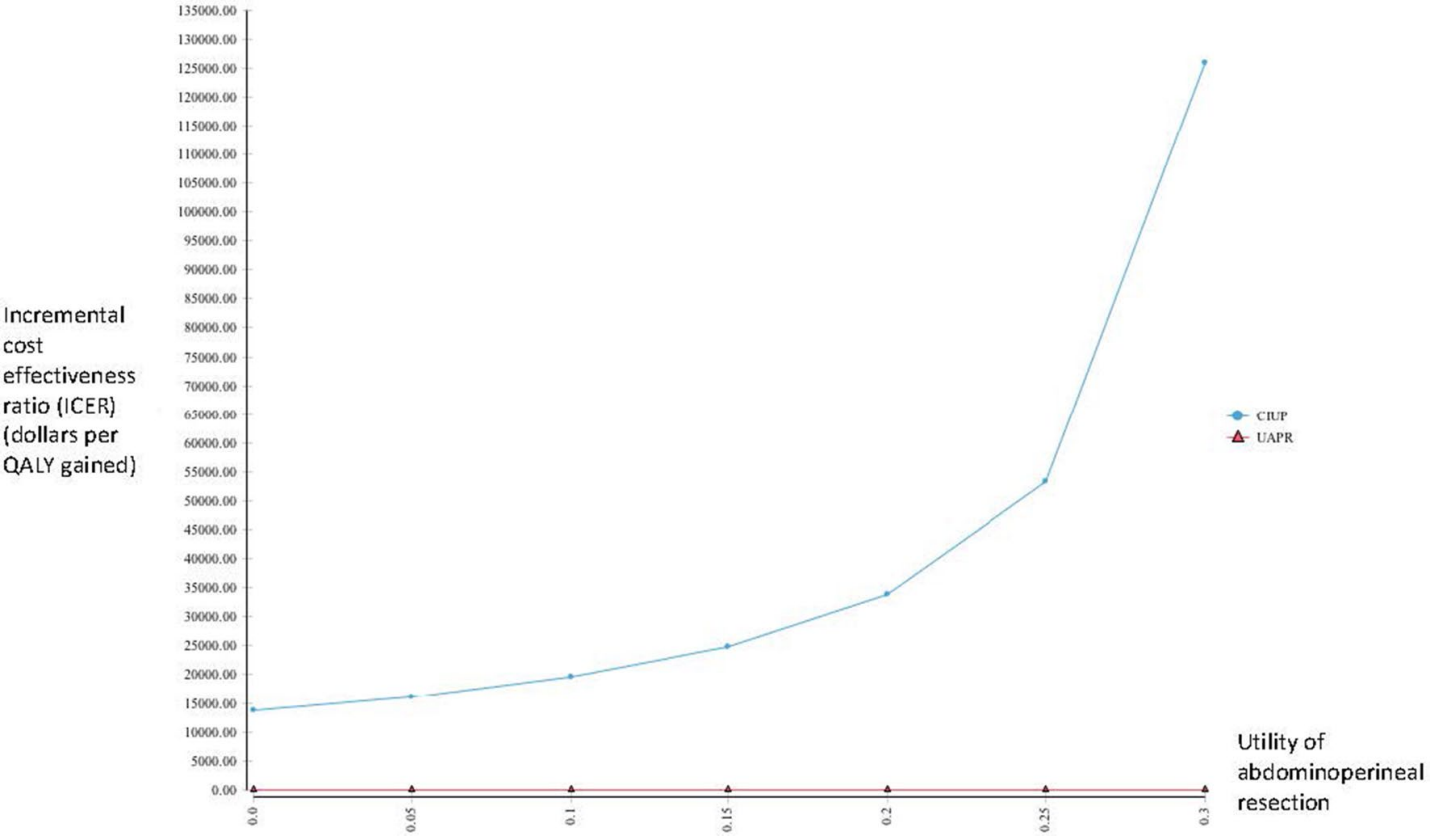
One way sensitivity analysis evaluating incremental cost effectiveness ratios (ICERs) of UAPR compared with CIUP for a range of utilities associated with abdominoperineal resection

**Fig. 3:**
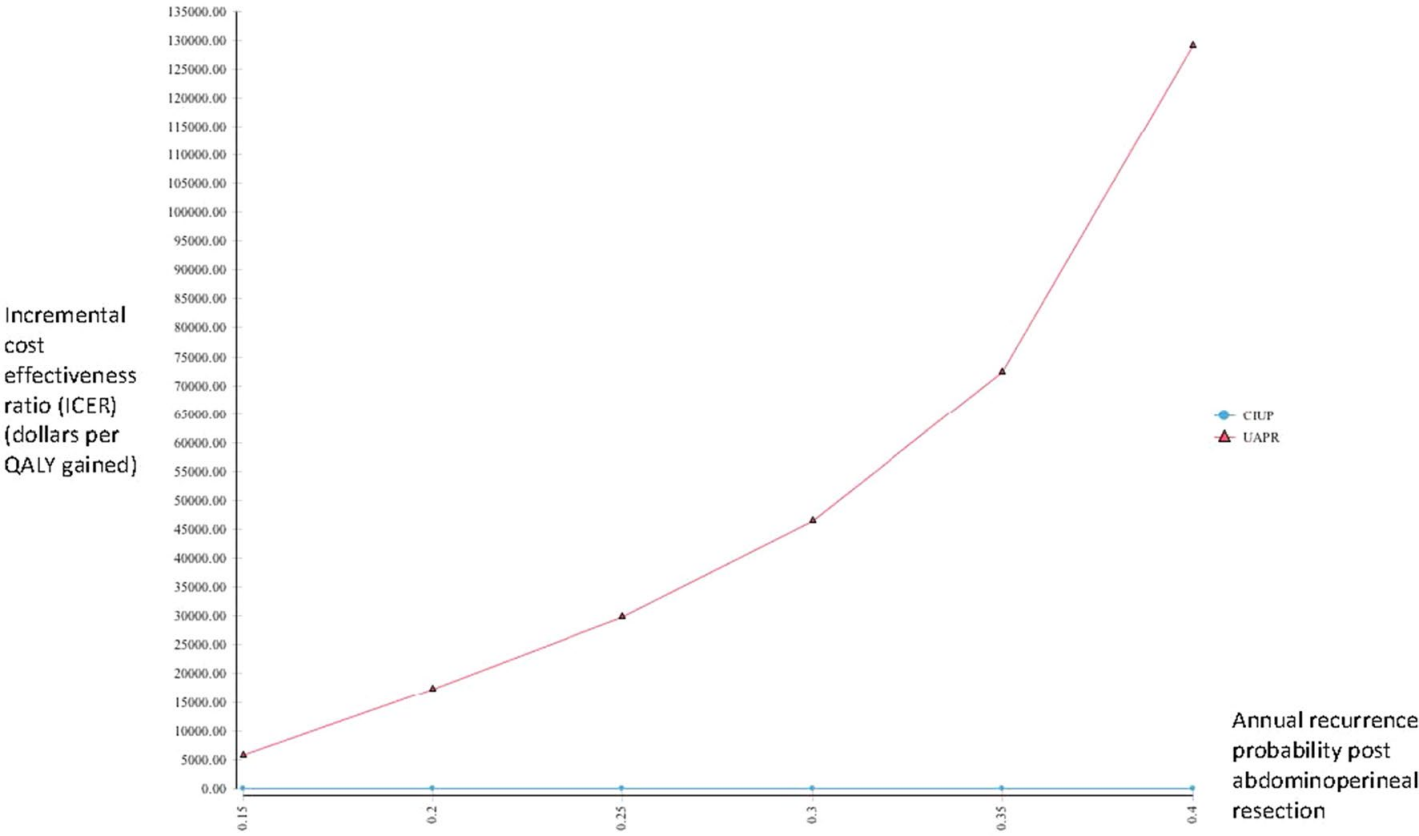
One way sensitivity analysis evaluating incremental cost effectiveness ratios (ICERs) of UAPR compared with CIUP for a range of annual recurrence probabilities post abdominoperineal resection. Below a recurrence probability of 12%, UAPR dominates CIUP–it costs less and is more effective, thus generating no meaningful ICER. The range of recurrence probabilities considered thus begins with 15%

**Fig. 4:**
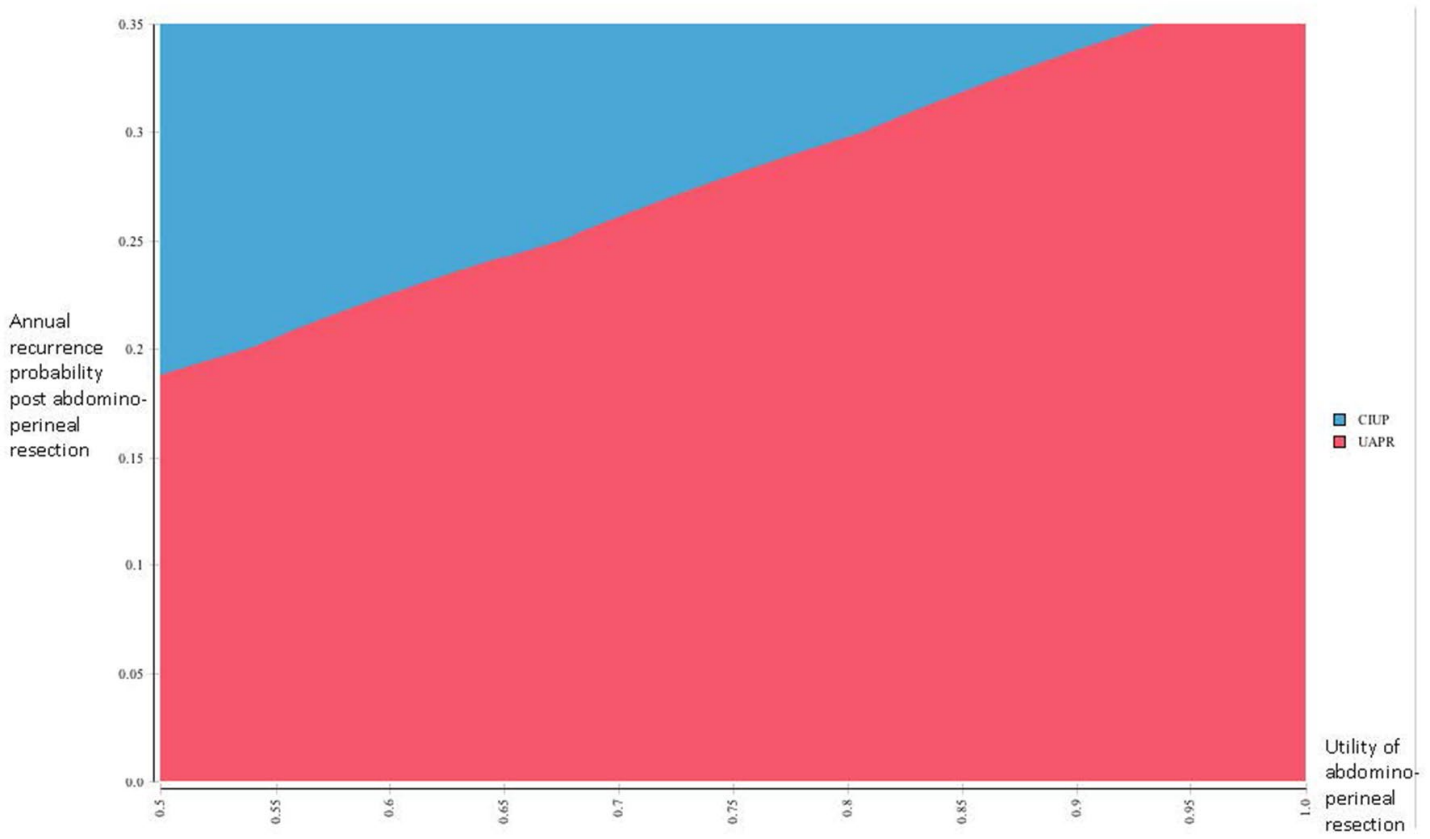
Two way sensitivity analysis comparing the net monetary benefit (NMB) of UAPR vs CIUP at a willingness to pay of SGD 50,000 when simultaneously considering varying values of utility associated with abdominoperineal resection and annual recurrence probabilities post abdominoperineal resection. Red denotes UAPR having superior NMB, while blue denotes CIUP having superior NMB

We performed several scenario analyses to further delineate the impact of extreme values for several variables on our outcome. When considering extremely low values of utility for the abdominoperineal resection, CIUP generated more QALYs than UAPR for utility values below 0.34, with CIUP producing ICER less than SGD 50,000 for utility values less than 0.24 (Fig. 2 ). As earlier described, UAPR ceased to dominate CIUP when post-abdominoperineal resection recurrence exceeded 12%. Extending this analysis to consider even higher annual probabilities of recurrence post abdominoperineal resection, UAPR generated more QALYs for annual recurrence probabilities below 47.0%; for higher annual recurrence probabilities, CIUP became the dominant strategy. UAPR remained more cost-effective than CIUP at WTP of SGD 50,000 for annual recurrence probabilities below 31% (Fig. 3). Additionally, we performed a 2-way sensitivity analysis exploring extreme values of utility for abdominoperineal resection (as low as 0.5) and annual recurrence probabilities post abdominoperineal resection (as high as 35%). In this analysis, the net monetary benefit for UAPR would always be superior for annual recurrence probabilities lower than 18% (Fig. 4).

**Fig. 5:**
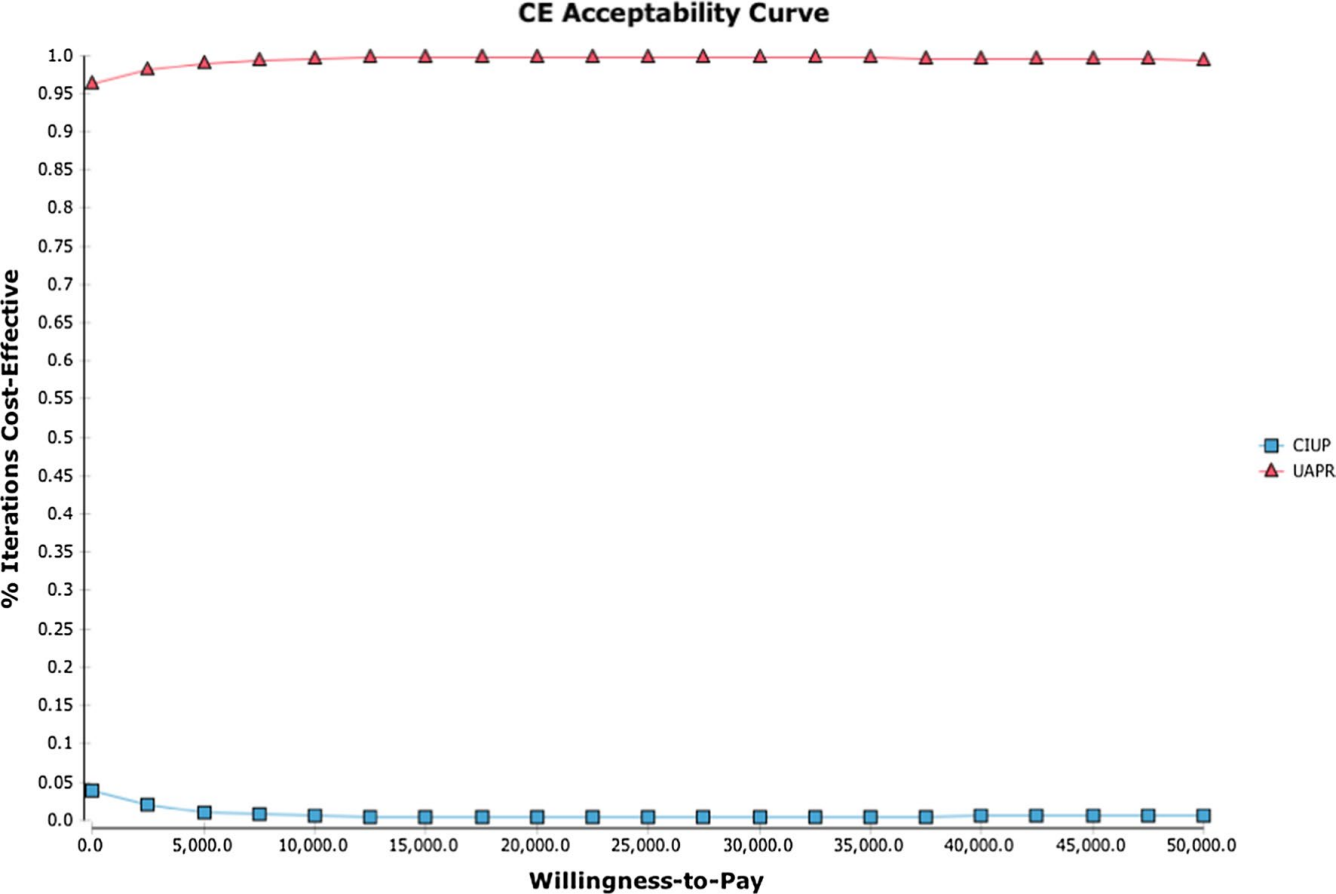
Cost effectiveness acceptability curve from probabilistic sensitivity analysis comparing UAPR and CIUP. UAPR has a 100% probability of being more cost effective that CIUP for willingness to pay of SGD 10,000 and above

From the PSA, the cost-effectiveness acceptability curve is shown in Fig. 5.

The probability of UAPR being cost-effective compared with CIUP is 100% for WTP SGD 10,000 and above, and never drops below 95%. Consistent with the findings from the base case analysis, the cost-effectiveness plane reveals UAPR to be dominant over CIUP for the majority of iterations of the PSA at a WTP of SGD 50,000.

## Discussion

In this cost-effectiveness analysis, considering rectal GIST patients with no disease progression following 12 months of neoadjuvant imatinib who required abdominoperineal resection for surgical extirpation, UAPR is more effective and less costly than CIUP. These results remained robust after considering one-way DSA of multiple factors. The only exception to this arose when the annual probability of recurrence following abdominoperineal resection exceeded 12.5%; in this situation, UAPR was costlier and more effective than CIUP, with ICER values remaining well below the SGD 50,000 WTP threshold. These findings were replicated in PSA, with the probability of UAPR being cost-effective compared with CIUP being 100% from a WTP threshold of SGD 10,000 onwards. Taken in aggregate, our data suggest that, consistent with current practice, surgical extirpation must necessarily remain the primary curative treatment modality in localized rectal GIST. Such a conclusion would be consistent with the practice and evidence from the treatment of solid tumors in general [22], and GIST in particular [3]. Complete resection should thus not be forsaken in the treatment of rectal GIST.

To account for the possibility of widespread individual variation in utility of abdominoperineal resection, we performed one way scenario analyses to explore the impact of extreme aversion to abdominoperineal resection through evaluation of very low values of utility associated with abdominoperineal resection. We found that CIUP would be more cost effective than UAPR (WTP of SGD 50,000) only if utility values were 0.24 or lower. This hypothetical value, assigning extraordinarily severe disutility to abdominoperineal resection, is much lower than the value of 0.83 described in the literature [7], and may be argued to be implausible in clinical practice. Nonetheless, we think that this information may be useful as a quantitative threshold to guide individualized discussions between physicians and patients with extreme aversion to abdominoperineal resection following neoadjuvant imatinib for rectal GIST.

As earlier described, UAPR ceased to dominate CIUP for annual recurrence probabilities following abdominoperineal resection greater than 12.5%. When more extreme values of recurrence probabilities were evaluated in a scenario analysis, the ICER of UAPR remained below SGD 50,000 for recurrence probabilities lower than 31.5%. By comparison, the annual recurrence probability following resection after neoadjuvant imatinib is 8.7% [8]; even if a 50% uncertainty of this point estimate is considered as an estimate of a 95% confidence interval, the upper bound of this confidence interval would be 13.0%. Considering other data, the annual probability of recurrence for high risk GIST resected upfront followed by 3 years of adjuvant imatinib in the landmark prospective study of adjuvant imatinib was 6.9% [5], and the annual recurrence probability of recurrence amongst imatinib-treated rectal GIST patients in another retrospective study was 5.7% [23]. These data suggest it extremely improbably that annual recurrence rates post resection of rectal GIST treated with imatinib would be high enough for UAPR to be rendered cost-ineffective compared with CIUP.

Probing the sensitivity to these variables further, we simultaneously explored extreme values of both utility associated with abdominoperineal resection and annual recurrence probabilities post abdominoperineal resection in a 2-way sensitivity analysis. In this analysis, UAPR was shown to be the optimal strategy at a WTP of SGD 50,000 for all cases when post abdominoperineal resection recurrence was less than 18% annually, regardless of how low the utility associated with abdominoperineal resection was (considering utility values as low as 0.5). Even when considering the lower bound of the confidence interval for utility associated with abdominoperineal resection (0.65) [7], UAPR would remain more cost-effective for annual recurrence probabilities post abdominoperineal resection below 24%. Either way, these annual recurrence probabilities below which UAPR would be the optimal strategy are well beyond the upper limit of the confidence interval for annual recurrence probability of 13%, reinforcing the robustness of the result favoring the superiority of UAPR. In addition, notwithstanding the improbability of such high annual recurrence rates in clinical practice, these data would be useful in therapeutic discussions with clinicopathologically high risk rectal GIST patients expressing severe aversion to the morbidity associated with abdominoperineal resection. Formally quantifying individual preferences, and comparing it against quantitated boundaries of transition probabilities associated with particular strategies, will hopefully enable more informed and precise therapeutic decision making.

**Fig. 6:**
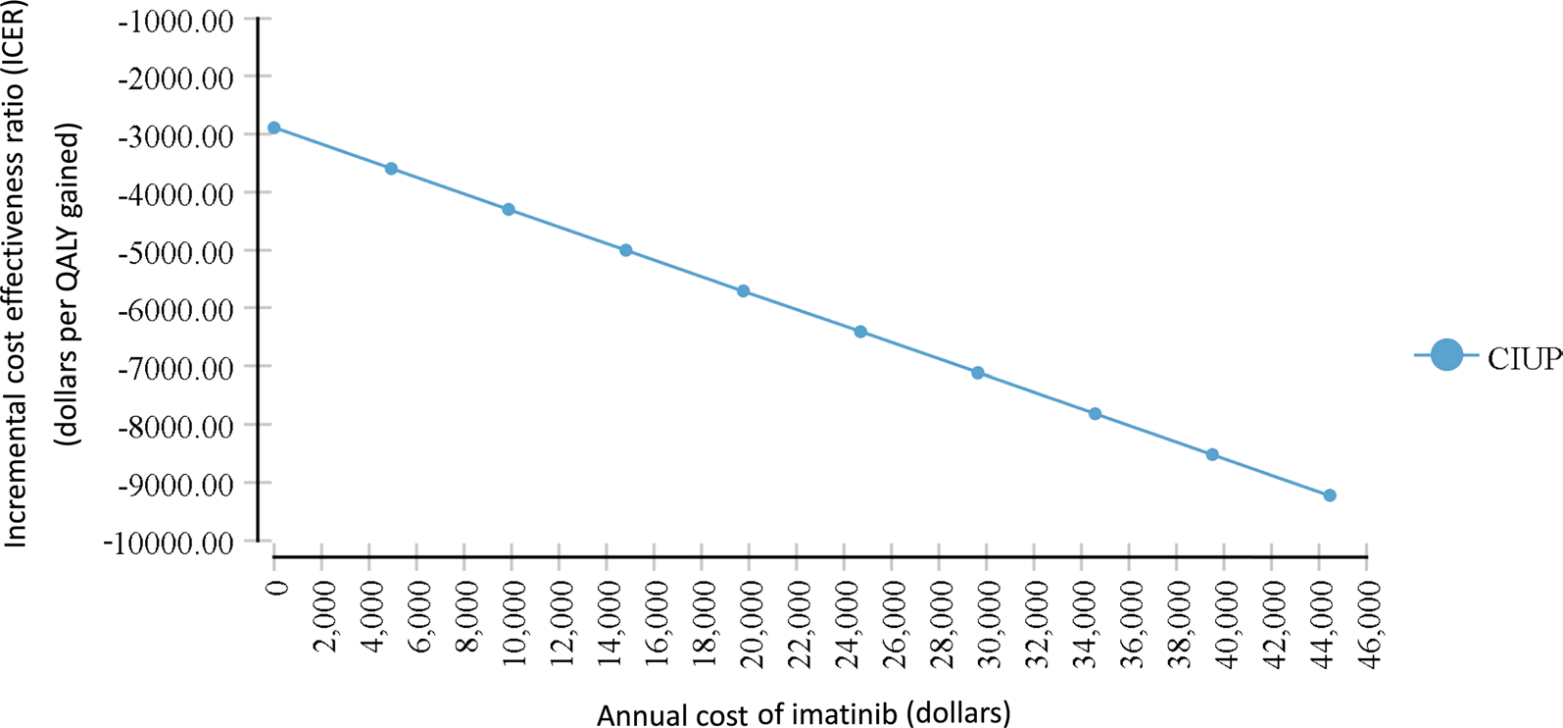
One way sensitivity analysis evaluating incremental cost effectiveness ratios (ICERs) of CIUP compared with UAPR for a range of costs of imatinib. For all values of imatinib price, no meaningful ICER is accrued. In the setting of the known finding of CIUP having less effectiveness, this suggests that it is the more costly strategy overall regardless of imatinib cost

The cost of imatinib was not a significant factor in determining the optimal strategy in this analysis. Since CIUP was less effective than UAPR, CIUP would not be more cost effective even if it was the less costly strategy. As it were, when cost effectiveness of CIUP relative to UAPR was evaluated in relation to how the cost of imatinib varied in a one-way sensitivity analysis, there was no

meaningful ICER accrued (the ICER remained negative) even when the cost of imatinib was zero dollars (Fig. 6). As ICER is calculated by dividing the difference in costs by the difference in effectiveness, and given that CIUP was less effective than UAPR, this suggests that the cost of the CIUP strategy was greater than the UAPR strategy, even if imatinib were free. This increased cost is likely related to the costs accruing from inferior clinical outcomes with CIUP.

There are several limitations to our study. Our analysis applies only to patients for whom abdominoperineal resection is required following neoadjuvant imatinib, a situation that applies to the minority of patients. In the imatinib era, with the excellent clinical responses and long term disease control afforded by imatinib, there are a range of less morbid surgical procedures available to patients without compromising completeness of surgical extirpation. In one of the largest series of rectal GIST reported, the rate of patients requiring APR or pelvic exenteration in the imatinib era was only 3%, compared with 59% before the advent of imatinib [24]. Nonetheless, our aim was to very specifically evaluate the outcomes for the most morbid surgical scenario—loss of bowel continence—to see if deferring surgery could possibly be the more cost-effective option following neoadjuvant imatinib, an option that patients faced with such a clinical scenario may be inclined to consider (as distinct from the situation when surgery has less morbidity, and thus opting for upfront surgery is more clear-cut). As it turns out, our analysis confirms the importance of complete surgical extirpation even if surgical morbidity is a certainty. We did not consider the utilities and costs of toxicity accruing from drug therapy, primarily because the toxicity from imatinib is generally limited and tolerable [25]. Additionally, drug toxicities from sunitinib and regorafenib would only become relevant in the setting of advanced disease, and would not differentially affect costs and effectiveness across the 2 strategies. We did not consider indirect costs such as loss of earnings from disrupted employment or costs to unpaid caregivers, given that we employed a healthcare sectors’ perspective as opposed to a societal perspective in our analysis [26]. We did not include disease related prognostic factors like size, mitotic rate and mutational status in our model. This is because full clinicopathologic characterization (pathological size as well as mitotic count) is not available at the start of neoadjuvant imatinib for rectal GIST, where diagnosis is often based on endoscopically acquired fine needle biopsy —so these factors do not dictate initiation of imatinib in clinical practice. In addition, the primary value of mutational status is in ensuring only patients with imatinib sensitive mutations (*KIT* mutations, and *PDGFR*A mutations other than D842V) receive imatinib–this group forms the vast majority (> 80%) of GIST patients. There were, however, patient (surgical fitness, specific anatomical factors, comorbidities and resources for self-care, perceptions of surgical morbidity) and treatment (surgical expertise) prognostic factors that we did not account for. It may be argued that a WTP of SGD 50,000 is too conservative for contemporary considerations of cost-effectiveness [17]; however, given the robustness of our results, the consideration of higher levels of WTP is not likely to alter our conclusions.

## Conclusions

In summary, our cost-effectiveness analysis confirms the importance of surgical extirpation in rectal GIST patients who have received neoadjuvant imatinib, showing upfront abdominoperineal resection to be more effective and less costly than continued imatinib until progression. These results remain robust after both deterministic and probabilistic sensitivity analysis of various factors influencing both cost and effectiveness of the competing strategies. In aggregate, these results may be useful to guide personalized clinical discussions between physicians and patients considering potentially morbid surgery after a period of neoadjuvant therapy in rectal GIST, especially in patients with a strong aversion to surgical morbidity from abdominoperineal resection.

## Data Availability

All data generated and/or analyzed during this study are included in this published article.

## Abbreviations

GIST: Gastrointestinal stroma tumor
SGD: Singapore dollars
UAPR: Upfront abdominoperineal resection
CIUP: Continued imatinib until progression
QALY: Quality adjusted life years
WTP: Willingness to pay
PFS: Progression free survival
OS: Overall survival
ICER: Incremental cost effectiveness ratio
NCCS: National Cancer Centre Singapore
CT: Computed tomography
DSA: Deterministic sensitivity analysis
PSA: Probabilistic sensitivity analysis
